# Do All Switches Cost the Same? Reliability of Language Switching and Mixing Costs

**DOI:** 10.5334/joc.140

**Published:** 2021-01-07

**Authors:** Dorit Segal, Anat Prior, Tamar H. Gollan

**Affiliations:** 1 University of California, San Diego, US; 2 University of Haifa, Israel

**Keywords:** reliability, variability, language switching, color-shape switching, multi-tasking, bilingualism

## Abstract

The current study examined the reliability and consistency of switching and mixing costs in the language and the color-shape tasks in three pre-existing data sets, to assess whether they are equally well suited for the study of individual differences. Specifically, we considered if the language task is as reliable as the color-shape task – an important question given the wide use of language switching tasks but little information available to address this question. Switching costs had low to moderate reliability and internal consistency, and these were similar for the language and the color-shape tasks. Mixing costs were more reliable in the language task than in the color-shape task when tested twice on the same day and trended in the same direction when tested a week apart. In addition, mixing costs were larger and more consistent than switching costs in all data sets and they were also were more reliable than switching costs in the language task when tested on the same day. These results reveal the language task to be as good as the color-shape task for measuring switching and mixing ability. Low variability of switching costs may decrease their reliability and consistency, in turn interfering with the chance of detecting cross task correlations. We advocate for exploring procedures to increase the variability of switching costs, which might increase reliability and consistency of these measures, and improve the ability to determine if bilingual language use relies on cognitive mechanisms that overlap with those underlying nonlinguistic multi-tasking.

One of the most extraordinary aspects of the human mind is the ability to execute two concurrent tasks – people can walk and talk at the same time or listen to music while reading a book. In other situations, multitasking is extremely difficult or even impossible – people cannot read a book while cooking and cannot speak two languages at the same time. In these situations, one needs to choose one task at the time and then switch tasks. In fact, multitasking can be viewed as a continuum in terms of the time spent on one task before switching to the other. On one end of the continuum is *concurrent multitasking* – tasks that are performed at the same time (e.g., driving and talking) and on the other end is *sequential multitasking* – tasks that require more time before switching them (e.g., speaking 2 languages; Salvucci, Taatgen, & Borst, 2009).

Many studies on sequential multitasking focused on the specificity of switching and tried to examine if there is one general mechanism for switching tasks in different domains (domain-general) or does each domain have its own switching mechanism (domain-specific). This question has received much attention in the literature on bilingualism. Bilinguals seem to easily switch languages at will, while also preventing unwanted switches. The extent to which this ability is specific to juggling languages (domain-specific), or reflects a more general switching ability, has been the focus of an ongoing heated debate (Paap et al., 2017; Prior & MacWhinney, 2010). One of the main approaches to address this question has been to examine whether bilinguals perform similarly on language switching and on non-linguistic (e.g., color-shape) switching tasks (Prior & Gollan, 2011). One can argue that these tasks are not comparable, since they fall upon different points on the multitasking continuum: Color and shape are usually processed simultaneously (e.g., a yield sign gets its meaning from processing its color together with its shape), while languages are produced sequentially (see also Segal, Stasenko, & Gollan, 2019). Note however, that in the experimental design, both tasks are sequential in nature. That is, in both tasks, participants perform one task (use one language or name one aspect of visual stimuli) and very soon after, they see a predefined cue and switch to the other task. In this sense, these tasks are on the same location on the multitasking continuum and can be compared.

For both language and non-linguistic tasks, switching ability is usually measured as the difference in response time to similar (stay) versus different (switch) consecutive trials (i.e., switching cost) in mixed blocks. The ability to monitor conflict between tasks and keep two task sets partially activated is measured as the difference in response time between stay trials in mixed blocks and single trials in a single-task block (i.e. mixing cost). The basic assumption is that if language and non-linguistic tasks share a common switching component, then individuals who excel in one task should also excel in the other task (e.g., good language switchers are also good task switchers) and these two abilities should correlate and show convergent validity.

Indeed, some studies found positive correlations between linguistic and non-linguistic switching (Declerck, Grainger, Koch, & Philipp, 2017; Timmer, Calabria, Branzi, Baus, & Costa, 2018). For example, Gollan, Kleinman, and Wierenga (2014) found that bilinguals, who often failed to switch between languages on a cued switching task also failed more often to switch between reading numbers aloud versus adding their digits. They also found that bilinguals who voluntarily switched languages often also chose to switch between reading and adding more often. Likewise, Prior and Gollan (2011) found that Spanish-English bilinguals, who switch languages frequently, showed smaller task switching costs than monolinguals and Segal et al. (2019) found a correlation between language and task switching when participants responded quickly. Weissberger, Wierenga, Bondi, and Gollan (2012) also found that older bilinguals who could not complete the color-shape task exhibited larger language-switching costs than matched bilinguals who were able to do both tasks. While these findings imply that language and task switching tap the same mechanism, supporting the existence of a domain-general switching mechanism, other studies report quite different results. For example, there is evidence of age-related decline in non-linguistic task switching but not in language switching tasks, and switching costs for a linguistic and a non-linguistic task were not correlated in young (Timmer, Calabria, & Costa, 2019), middle-aged or older bilingual adults (Calabria, Branzi, Marne, Hernandez, & Costa, 2015). These findings directly conflict with those presented above, and imply the opposite conclusion, i.e., that language switching is supported by language-specific switching mechanisms (See also de Bruin, Treccani, & Della Sala, 2015; Paap & Greenberg, 2013 and Paap, Johnson, & Sawi, 2015 for similar conclusions).

Most researchers acknowledge the importance of comparing tasks with similar designs, but many do so without considering another factor that can be critical for such comparisons – the reliability of the measures. An experimental measure that has poor reliability does not accurately measure the theoretical construct it is supposed to measure, and therefore, such a measure has only limited ability to detect relationships with other constructs. If people who show large switching costs on one day, show small switching costs on another day, it means that the task does not necessarily measure switching ability and the chances of detecting cross task correlations with other switching tasks decrease. Reliability affects mostly correlational studies because a correlation between measures will always be lower than the reliability of the measures (Cohen, Cohen, West & Aiken, 2003).

Reliability refers to the consistency of a measure either over time (test-retest reliability), across different items or trials within a single session (internal consistency), and across different researchers (inter-rater reliability). It measures the signal (variance in true score) to noise (measurement error) ratio in the data \left({True\ score\ variance} \over {{\rm{Measurement}}\ error\ varience} \right) (Matheson, 2019). While reliability of 1 means that all variability is accounted for by true differences (no measurement error), reliability of 0 means that all variability is attributed to measurement error. Measurement error can be further divided into error variance and the variance between sessions. Error variance is different for each participant and is measured by the standard error of participants’ mean. Increasing the number of trials, for example, can decrease the error variance and this in turn will increase reliability. The variance between sessions is related to more systematic changes between sessions. Measurement error, especially in measures of reaction time, is more likely to affect between than within session reliability, since RT measures may be strongly affected by changes in arousal, motivation and attention, which are more likely to change over days than within a single testing session. However, assessing test-retest reliability across sessions is valuable, since cognitive measures are often used to evaluate cognitive abilities at different points in time (or to compare between groups of individuals; see Paap & Sawi, 2016 for a detailed description). Assuming constant measurement error, reliability increases when true-scores vary a lot between individuals. In turn, this makes it easier to accurately rank individuals by ability, and also to detect any existing relationships with other measures.

The color-shape switching paradigm has been shown to have acceptable reliability and consistency. For example, Paap and Sawi (2016) examined the test-retest reliability of a few commonly used neuropsychological tasks administered on two different days (tested a week apart) in 75 monolinguals and bilinguals of various language combinations. One of them was the color-shape task, for which they report relatively high test-retest reliability for mean RTs in switch, repeat and single trials (0.86, 0.87 and 0.77, respectively), and lower reliability for differences between them (0.62 for switching costs, and 0.75 for mixing costs). Timmer et al. (2018) examined the test-retest reliability, tested over a week, of a non-linguistic task, in which participants had to switch between three perceptual classifications of visual stimuli: ‘color’ (red vs. blue), ‘size’ (small vs. big), and ‘type’ (letter vs. number). They found reliability of 0.57 for proportional switching costs (switching costs divided by the average of switch and stay trials). Von Bastian, Souza, and Gade (2016) examined the internal consistency (split half) of three non-linguistic switching tasks (Color-shape, Animacy-size and Parity-magnitude) and found extremely high consistencies: 0.91 for switching and 0.96 for mixing costs (proportional costs averaged across tasks). In a later study, von Bastian and Druey (2017) found consistency of 0.79 for log transformed switching costs in the color-shape task.

In contrast to the non-linguistic tasks and despite of their common use, only few studies have thoroughly considered the reliability and the consistency of language switching tasks. To our knowledge, the only study that compared the test-retest reliability of linguistic versus non-linguistic switching tasks was Timmer et al. (2018), who found strong test-retest reliability in language switching costs (0.739). However, this study investigated trilinguals naming pictures in three languages, whereas most language switching studies examine bilinguals using two languages. In addition, Timmer et al.’s set up (looking at n-1 switching costs and n-2 repetition costs) caused an unequal distribution of stay and switch trials (about 70 stay and 250 switch trials). In a second study, Contreras Saavedra, Koch, Schuch, and Philipp (2020) examined the internal consistency (correlating even and odd trials) of a language-switching task, in which participants named single digit numbers, and double-digit numbers, switching between English and German. The task included an equal distribution of stay and switch trials. They found reliability of 0.71 for standard switching cost and 0.64 for proportional switching cost.

Differences across language switching paradigms in the nature of response sets, response modality, the number of trials, and the proportion of trials of different types (how often participants repeat and switch tasks) could have critical effects on the magnitude of switching costs, and likely also on test-retest reliability (Bonnin, Gaonac’h, & Bouquet, 2011; Contreras Saavedra et al., 2020; Schneider & Logan, 2006). In addition, mixing costs were not measured either in Timmer et al.’s or in Contreras Saavedra et al.’s study, although mixing costs have often demonstrated more consistent correlations across linguistic and nonlinguistic domains than switching costs (Prior & Gollan, 2013; Segal et al., 2019; Stasenko, Matt, & Gollan, 2017; Timmer et al., 2019).

Thus, in the current study we set out to assess the reliability and consistency of language switching using the parameters most common in the bilingual language switching literature. To this end, we examined test-retest reliability and the internal consistency (correlation between even and odd trials) of switching and mixing costs in the language switching task and compared them to the reliability and consistency of the color-shape switching tasks in three existing data sets. We addressed two main questions: Are language switching and mixing costs reliable across and within sessions? Is the reliability of language switching and mixing costs comparable to that of the commonly used color-shape task?

## METHODS

### PARTICIPANTS

As shown in ***Table 1*** and ***Table 2***, data from bilingual participants in 3 different studies were analyzed: 116 bilinguals from Prior and Gollan (2013), 78 bilinguals from Stasenko et al. (2017) and 288 bilinguals from Kleinman and Gollan (2018). All three studies included young adult participants, who used two languages in their daily lives. Language combinations were Spanish-English, Mandarin-English and Hebrew-English. In all three studies participants performed a language switching task, and in the first two they also performed a non-linguistic (color-shape) switching task.

**Table 1:** Methodological details of the 3 studies.


	PRIOR AND GOLLAN (2013)	STASENKO ET AL. (2017)	KLEINMAN AND GOLLAN (2018)

Participants	116 bilinguals (4 were excluded)^a^	78 Spanish-English bilinguals (2 were excluded)^a^	288 Spanish-English bilinguals

Number of sessions	Two – a week apart	One	One

Task order	Session 1 – language history questionnaire, two similar experimental tasks (language *or* color-shape) Session 2 – two different experimental tasks (language *and* color shape)^b^ and even items of MINT^c^	Language history questionnaire, color-shape switching, language switching, color-word interference test, trail making test, and MINT^c^	Language switching, language history questionnaire, and the MINT^c^

Experimental tasks and response type	Language switching (digits) – spoken responses Color-shape switching – spoken responses	Language switching (digits) – spoken responses Color-shape – button press	Language switching (picture naming) – spoken responses.

Number of trials per condition	160 trials: 80 single trials (4 blocks) and 80 mixed trials (4 blocks of 20 trials) in sandwich design Switch rate: 50%	480 trials (half short, 116 ms, and half long, 1016 ms Cue-Target Interval (CTI): 160 single, ~ 160 stay and ~ 160 switch trials. Switch rate: 53%	324 trials: 216 single trials (2 blocks of 108 trials) and 108 mixed trials (1 block). Switch rate: 33%

Reliability analyses	Test retest Internal consistency (even-odd comparisons)	Internal consistency (even-odd comparisons)	Internal consistency (even-odd comparisons)


^a^ To maximize statistical power we included all participants tested in Prior and Gollan (2013; without excluding 9 Spanish-dominant and 12 Chinese-dominant bilinguals). There were 30 Hebrew-English, 29 Chinese-English bilinguals, and 61 Spanish-English, for a total of 120 participants (four participants were trimmed so that the final sample included 116 participants). In Stasenko et al. (2017), two participants were excluded. ^b^ Half of the participants completed (only) the language task twice in the first session (Training 1 and 2) and once again (Training 3), after completing the color-shape task (transfer task), a week later (hereafter, the *language training group*). The other half completed (only) the color-shape task twice in the first session and once again, after completing the language task a week later (hereafter, the *color-shape training group*). ^c^ Multilingual Naming Test (Gollan et al., 2012).

**Table 2:** Participant characteristics in Prior and Gollan (2013)^a^.


	HEBREW-ENGLISH^b^ (N = 30)	MANDARIN-ENGLISH (N = 29)	SPANISH-ENGLISH (N = 61)

Age	25	20	20

English self-rated proficiency	5.8	5.9	6.5

Other language self-rated proficiency	7	5.4	6.0

English MINT^c^	24.4	28.8	29.3

Other language MINT	31.6	25.8	23.8

Primary caregiver yrs education	15.9	15.4	10.9

Secondary caregiver yrs education	14.7	15.7	10.3

Participant yrs education	13.4	13.2	13.9

English percentage daily use	12.4^a^	79.9	79.6

Age of first exposure to English (yrs)	8.1	5.1	4.2


^a^ Note that we only describe participant characteristics from Prior and Gollan (2013), and not from the other data sets, because the sample we analyzed herein was substantially different from the original study (i.e., to maximize power in the present study we included all bilinguals including late-learners and those not dominant in the majority language). Language proficiency was rated on a 1 to 7 scale. Ratings presented here are averaged across speaking, listening, reading and writing. ^b^ One participant in this group did not report daily percentage of English use. ^c^ Based on half of the MINT items.

### MATERIALS AND PROCEDURE

***Table 1*** briefly describes the tasks and methods of Prior and Gollan (2013), whose data were analyzed for test-retest reliability (on the same day and one week apart) and for internal consistency (correlations between even and odd trials within one session). Internal consistency was measured in the first administration of each task in each group separately and was compared to the internal consistency of Stasenko et al. (2017), and Kleinman and Gollan (2018), whose data were only analyzed for internal consistency, because participants in these studies were tested in just one session (See ***Table 1*** for a brief description of these studies and ***Table 3*** for a more detailed description of the study design of Prior & Gollan, 2013).

**Table 3:** Study design (of Prior & Gollan, 2013).


	LANGUAGE SWITCHING		TASK SWITCHING

Blocks 1–2	Single-language blocks (1 English & 1 other, order counterbalanced)		Single-task blocks (1 color & 1 shape, order counterbalanced)

Blocks 3–6	4 mixed English/other blocks		4 mixed color/shape blocks

Blocks 7–8	Single-language blocks (1 English & 1 other, order reversed from blocks 1 & 2)		Single-task blocks (1 color & 1 shape, order reversed from blocks 1 & 2)

**COUNTERBALANCING OF TRAINING AND TRANSFER SEQUENCES**			

***TIME POINT***	***TRAINING CONDITION***	***LANGUAGE TRAINING GROUP***	***COLOR-SHAPE TRAINING GROUP***

Day 1	Training 1	Language switching	Color-shape switching

	Training 2	Language switching	Color-shape switching

Day 2	Transfer	Color-shape switching	Language switching

	Training 3	Language switching	Color-shape switching


## RESULTS

The three data sets were trimmed in the same way to enable comparisons^1^. As in Paap and Sawi (2016), accuracy rates in all studies were extremely high (above 96%) and therefore, we focused on RT measures only. ***Table 4*** presents the means and SDs of RTs in the 3 data sets, after the trimming procedure, by trial type. ***Table 5*** presents the test-retest reliability (Pearson correlation) measured on the first and second administrations of the same day (training 1 and 2) and a week apart (training 2 and 3) and the internal consistency (Pearson correlation between even and odd trials) of single, stay and switch trials and of the switching and mixing costs of the language switching task in Prior and Gollan (2013), as well as the internal consistency of these measures in Stasenko et al. (2017), separated by CTI, and in Kleinman and Gollan (2018). ***Table 6*** presents similar data, but for the color-shape task (note that there was no color-shape task in Kleinman & Gollan). The main findings are summarized in ***Table 8***.

**Table 4:** Means and SDs of the different trial types and the switching and mixing costs across tasks in the language and the color-shape tasks.


	PRIOR & GOLLAN, 2013				STASENKO ET AL., 2017				KLEIMAN & GOLLAN, 2018	
	
	1^ST^ SESSION^a^		TRANSFER TASK^b^		CTI LONG		CTI SHORT			
				
	M	SD	M	SD	M	SD	M	SD	M	SD

***LANGUAGE***										

Single	505	64	509	51	520	68	549	75	689	76

Stay	554	86	550	75	600	114	675	120	796	107

Switch	575	97	577	90	644	127	728	132	841	125

Switch cost	21	30	28	30	44	38	53	34	45	44

Mix cost	49	50	40	38	81	68	127	68	106	66

***COLOR-SHAPE***										

Single	541	69	569	103	526	117	546	122		

Stay	601	85	565	214	686	213	920	256		

Switch	629	89	679	202	708	217	980	251		

Switch cost	28	30	25	32	22	51	61	69		

Mix cost	60	46	73	65	160	147	374	186		


^a^ First administration of the task on the first day. ^b^ Administration of the task on the second day after training in the other task.

**Table 5:** Test-retest reliability of single, stay and switch trials and of switching and mixing costs in the language switching task in Prior and Gollan (2013) and internal consistency (correlations between even and odd trials) of the language switching task by study.


	TEST-RETEST		INTERNAL CONSISTENCY				
	
	PRIOR AND GOLLAN (2013)		PRIOR AND GOLLAN (2013)		STASENKO ET AL. (2017)		KLEINMAN & GOLLAN (2018)
		
	SAME DAY	OVER A WEEK	1^ST^ SESSION^a^	TRANSFER TASK^b^	CTI LONG	CTI SHORT	

Single	0.92	0.87	0.98	0.97	0.96	0.97	0.97

Stay	0.93	0.88	0.93	0.96	0.97	0.97	0.93

Switch	0.92	0.82	0.95	0.96	0.96	0.97	0.90

switching cost	0.53	0.52	0.32	0.45	0.37	0.41	0.22

mixing cost	0.79^*c^	0.67	0.77*	0.79*	0.89*	0.87*	0.81*


^a^ First administration of the task on the first day. ^b^ Administration of the task after training on the other task ^c^ The only significant difference across domains (i.e., comparing analogous values shown in Tables 5 and 6). * Significantly different from the cell above it (*p* < .01). ^#^ n.s (*p* > .05).

**Table 6:** Test-retest reliability of single, stay and switch trials and of switching and mixing costs in the color-shape switching task in Prior and Gollan (2013) and internal consistency (correlations between even and odd trials) of the color-shape switching task by study.


	TEST-RETEST		INTERNAL CONSISTENCY			
	
	PRIOR AND GOLLAN (2013)		PRIOR AND GOLLAN (2013)		STASENKO ET AL. (2017)	
		
	SAME DAY	OVER A WEEK	1^ST^ SESSION^a^	TRANSFER TASK^b^	CTI LONG	CTI SHORT

single	0.92	0.87	0.97	0.97	0.96	0.95

stay	0.88	0.82	0.92	0.94	0.96	0.97

switch	0.90	0.76	0.91	0.95	0.96	0.97

switching cost	0.56	0.40	0.14^#^	0.29	0.17^#^	0.43

mixing cost	0.53^c^	0.51	0.70*	0.82*	0.91*	0.91*


^a^ First administration of the task on the first day. ^b^ Administration of the task after training on the other task. ^c^ The only significant difference across domains (i.e., comparing analogous values shown in Tables 5 and 6). * significantly different from the cell above it (*p* < .01). ^#^ n.s (*p* > .05).

**Figure 1:**
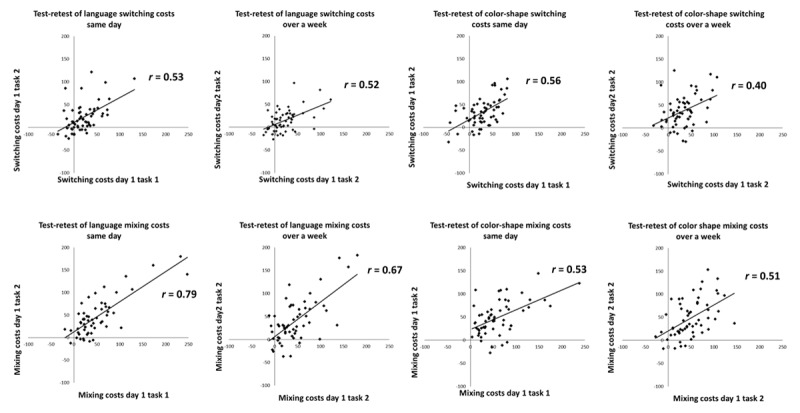
Test-retest reliability of language and color shape switching and mixing costs in Prior and Gollan (2011) when tested on the same day and a week apart. Switching costs in the top row, mixing costs in the bottom row.

**Figure 2:**
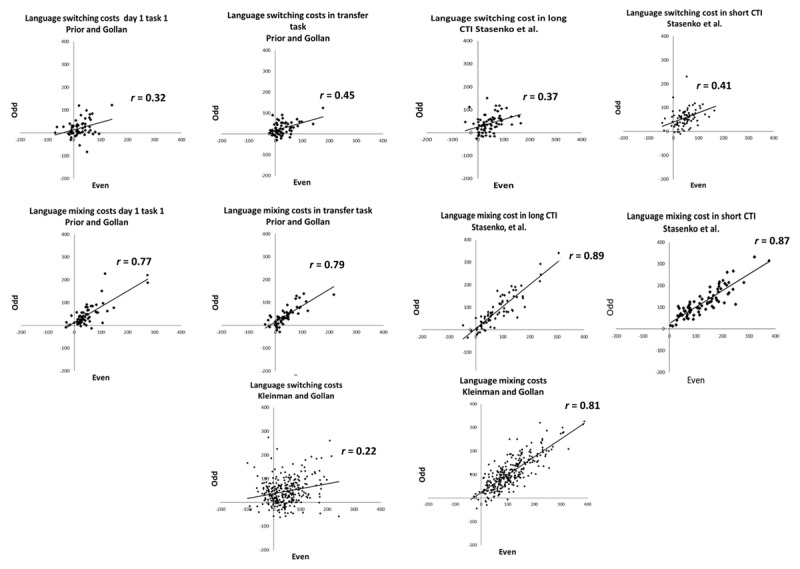
Internal consistency (i.e., correlating even and odd trials) of language switching and mixing costs across studies.

**Figure 3:**
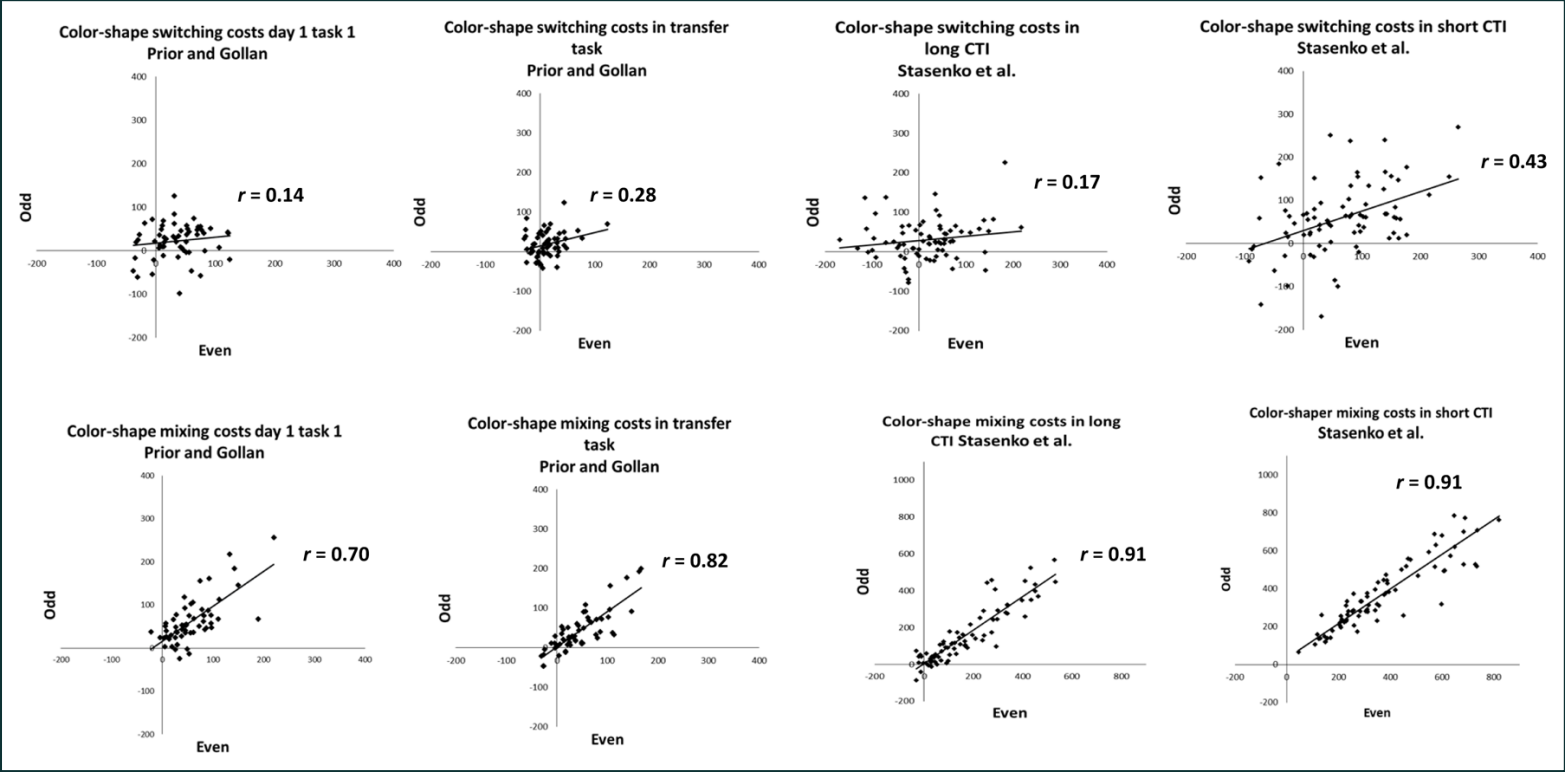
Internal consistency (i.e., correlating even and odd trials) of color-shape switching and mixing costs across studies (n.b., the axes for mixing costs in Stasenko et al. were adjusted for short and long CTI).

***Figure 1*** presents test-retest reliability (on the same day within a single testing session and a week apart) of the language and the color-shape switching tasks in Prior and Gollan (2013; but to maximize power also including all the bilinguals who were not English-dominant, which Prior & Gollan excluded). ***Figure 2*** presents the internal consistencies of the language-switching task in the three data sets; ***Figure 3*** presents the internal consistencies for the color-shape switching task.

### TEST-RETEST RELIABILITY AND INTERNAL CONSISTENCY OF LANGUAGE VERSUS COLOR-SHAPE SWITCHING AND MIXING COSTS

#### Switching Costs

As shown in ***Table 5*** (language task) and ***Table 6*** (color-shape task), test-retest reliability and internal consistency for switching costs were low to moderate. When comparing correlations within the same sample (single sided testing; Lenhard & Lenhard, 2014), there was no difference between reliability when tested twice on the same day versus when tested a week apart for both the language task, (*r* = .53 versus *r* = .52 respectively, *z* = .03, *p* = .972) and the color-shape task (*r* = .56 versus *r* = .40 respectively, *z* = .12, *p* = .232). Comparing across tasks, test-retest reliability of language and color-shape switching costs was similar on the same day (*r*_language_ = .53, *r*_color-shape_ = .56, *z* = .22, *p* = .822) and when tested a week apart (*r*_language_ = .52, *r*_color-shape_ = .40, *z* = .80, *p* = .424). The internal consistency of switching costs was also similar across both tasks in all studies (all *z*s < 1.33, all *p*s > .100).^2^

#### Mixing Costs

For the language task, test-retest reliability of mixing costs was moderate in size, and was marginally larger when tested on the same day than when tested a week apart (*r* = .79 versus *r* = .67; *z* = 1.91, *p* = .056). For the color-shape task, test-retest reliability of mixing costs were similar when tested on the same day and when tested a week apart (*r* = .53 versus *r* = .51; *z* = .18, *p* = .856; single-sided testing; Lenhard & Lenhard, 2014). Additionally, mixing costs were more reliable in the language task than in the color-shape task when tested twice on the same day (*r*_language_ = .79, *r*_color-shape_ = .53, *z* = 2.52, *p* = .012), and trended in the same direction when tested a week apart (*r*_language_ = .67, *r*_color-shape_ = .51, *z* = 1.30, *p* = .100). The internal consistency of mixing costs was similar for both tasks in all studies (all *z*s < 1.20, all *p*s > .230).

### TEST-RETEST RELIABILITY AND INTERNAL CONSISTENCY OF SWITCHING COMPARED TO MIXING COSTS

In the language task, when tested on the same day, mixing costs were more reliable than switching costs (*z* = –2.52, *p* = .010) and more consistent than switching costs in all data sets (all *z*s < 5.42, all *p*s < .002). However, when tested a week apart, language switching and mixing costs were equally reliable (*z* = –1.23, *p* = .219). In the color-shape task, switching and mixing costs were equally reliable when tested on the same day (*z* = .22, *p* = .820) and when tested one week apart (*z* = .73, *p* = .466), but mixing costs were more consistent than switching costs in all data sets (all *z*s < 7.20, all *p*s < .0001).

In addition, as shown in ***Table 4***, mixing costs were larger than switching costs in all three studies and the effect of trial type (single, stay, switch) was significant in both language and color-shape switching tasks (*p* < .001 for all comparisons). Mixing costs were also larger than switching costs within each task when comparing even to odd trials (p < .001 for all comparisons on both even and odd trials). As shown in ***Table 7***, an ANOVA with trial type and trial parity (even and odd) as within subject variables, showed a main effect of trial type but no interaction with parity, suggesting that the basic pattern of larger mixing than switching cost was similar for even and odd trials.

**Table 7:** ANOVA of the interaction between trial type and parity in language and color-shape tasks across studies.


	F	p	MSE

*Language*			

Prior & Gollan (1^st^ task)	<1	.898	353

Stasenko et al. (long)	1.33	.267	641

Stasenko et al. (short)	2.98	.060	346

*Color-shape*			

Prior & Gollan (1^st^ task)	2.68	.070	508

Stasenko et al. (long)	1.13	.325	1346

Stasenko et al. (short)	<1	.859	1585


**Table 8:** Summary of main findings.


	SWITCHING COSTS	MIXING COSTS

Comparing tasks	Same consistency and reliability across tasks.	Same consistency across tasks. Language more reliable than color-shape when tested twice on the same day and trending in the same direction when tested a week apart.

Day effects	No day effect: Similar reliability when tested on the same day and a week apart in both tasks.	No day effect: Similar reliability when tested on the same day and a week apart in both tasks.

Comparing mixing to switching costs	Mixing costs were larger and more consistent than switching costs in both tasks. Language task: Mixing costs were more reliable than switching costs when tested on the same day. Color-shape task: Mixing costs were as reliable as switching costs.	


## DISCUSSION

The current study examined test-retest reliability (testing twice on the same day, and a week apart) and internal consistency (comparing even to odd trials on the same day) of the commonly used language switching task and compared it to the reliability and consistency of the color-shape task. Test-retest reliability and internal consistency of language *switching costs* were low to moderate, were similar to the color-shape reliability and internal consistency and were less reliable and consistent than condition mean RTs (RTs on single, stay and switch trials). *Mixing costs* were more consistent than switching costs in all studies and in both tasks. Language mixing costs were more reliable than color-shape mixing costs when tested twice on the same day and trended in the same direction when tested a week apart. There was no difference in test-retest reliability of switching and of mixing costs when tested on the same day versus when tested a week apart in either task. Importantly, in spite of the relatively low consistency for switching costs, condition effects were remarkably consistent across studies, within each study (on even versus odd trials), and across linguistic and nonlinguistic domains. That is, mixing costs were larger than switching costs in every comparison.

### COMPARING LANGUAGE TO COLOR-SHAPE RELIABILITY AND CONSISTENCY

Language-switching costs were as reliable and as consistent as the color-shape switching cost, whereas language-mixing costs were generally more reliable than color-shape mixing costs. This suggests that participants’ ability to monitor a conflict between languages is more stable across administrations than their ability to monitor a conflict between color and shape. Bilinguals are used to monitoring two languages but not the arbitrary task-driven conflict between color and shape introduced in the experimental setting. This possibly led them to rely on the same mechanisms across different administrations of the language task, but to recruit different strategies across administrations of the color shape task. However, the fact that we report difference in the consistency of mixing costs between linguistic and non-linguistic tasks, but find comparable consistency in switching costs, might arise not only from differences in participants’ familiarity with the two tasks. Below we suggest that differences in the magnitude and nature of switching vs. mixing costs, as opposed to simple RTs, might also contribute to the reported pattern of results.

### RELIABILITY AND CONSISTENCY OF MEAN RTS COMPARED TO SWITCHING AND MIXING COSTS

Condition mean RTs in the current study on single, stay and switch trials were reliable, consistent and close to the criteria set by Miller and Ulrich (2013) in their IDRT model (0.9 for studies with more than 40 trials per condition). Switching costs on the other hand, and to a lesser extent mixing costs, were much less reliable and less consistent, a pattern also reported in previous research (Hedge, Powell, & Sumner, 2018; Paap & Sawi, 2016). We put forth two possible reasons for this finding.

The first explanation comes from the difference between general and specific components of performance. General components, such as processing of perceptual input and speed of motor output, are recruited by a wide variety of tasks, and are reflected in mean RTs. These processes must be shared across tasks and/or trials and/or administrations (e.g., participants who process information and respond quickly on one task are very likely to do so in a different task). Specific components, such as the flexibility required by switching, are only required in a specific experimental condition (e.g., switching component; for review see Kiesel et al., 2010). Because switching and mixing costs are difference scores, when subtracting performance in one condition from the other, the general processing components are eliminated and the remaining score in fact measures components that are specific to switching or to mixing. The more specific a component is, the less it is likely to be shared across tasks or administrations, and in the present case, will thus have lower reliability. Importantly, such difference scores are also more interpretable (See the individual differences in RT, IDRT model, by Miller & Ulrich, 2013 for more details).

A second possible reason why switching and mixing costs were less reliable and consistent than mean RTs is that in the current study switching and mixing costs were smaller and had lower variability, which compromises their utility in ranking individuals accurately and detecting relationships across tasks or administrations (Draheim, Tsukahara, Martin, Mashburn, & Engle, 2020).

### Comparing switching to mixing costs

The effect of the variability of a measure on its reliability can also explain why mixing costs in the current study were more consistent than switching costs. Mixing costs measure the difference in RTs between a single and a dual task, whereas switching costs measure a smaller difference between trials within the same block. The larger mixing compared to switching costs found across all studies, creates more room for variability (See figures and ***Table 4***) in mixing compared to switching costs. The larger variability allows for better ranking of mixing costs across sessions (Hedge et al. 2018) but also within a session, possibly increasing the consistency of mixing compared to switching costs. For example, in Stasenko et al. (2017) the mean mixing cost was 374 ms whereas the mean switching cost was only 61 ms in the color-shape task (short CTI condition) and the consistencies were 0.91 and 0.43, respectively. Note also that the larger mixing than switching costs found in all studies in both even and odd trials justifies the use of these measures for comparing costs across conditions (See also Segal et al., 2018).

However, mixing costs are more variable than switching costs, not just because of their relative sizes. For example, in Paap and Sawi (2016), switching costs were similar in size but less variable (*M* = 201, *SD* = 116 and *M* = 154, *SD* = 115 in first and second administrations respectively) than mixing costs (*M* = 218, *SD* = 258 and *M* = 133, *SD* = 185). Switching costs were also less reliable than mixing costs (0.62 vs. 0.74, respectively). Switching costs and mixing costs have been associated with different cognitive processes. Mixing costs are thought to reflect global processes of conflict monitoring and the need to keep two task sets partially activated whereas switching costs are thought to reflect the local, time-sensitive demands to allow inhibition of the previous task-set and activation of the currently relevant task and response set. The variability between individuals in conflict monitoring may be greater or more stable than differences between individuals in local management, which might also be more influenced by ongoing fluctuations in attention, and thus less stable. Indeed, in a previous study we found that switching costs, especially in the color-shape task, were more affected by lapses of attention or task uncertainty than mixing costs, even within a single session (Segal et al., 2018). This can also decrease the consistency of switching compared to mixing costs.

Note that the variability between individuals (needed for achieving high reliability) is crucial for detecting correlations, but it compromises the ability to detect group differences in experimental manipulations (Draheim et al., 2020; Hedge et al., 2018). Therefore, it is not surprising that many studies failed to find such correlations (Calabria et al., 2015), while observing concurrent group differences (Timmer et al., 2019). By contrast, the more variable mixing costs often do show cross task correlations (Prior & Gollan, 2013; Segal et al., 2018; Stasenko et al, 2017; Timmer et al., 2019). Many other cognitive tasks that use difference scores to measure a specific cognitive component also produce robust effects at the group level, but fail to show reliability as a measure of individual differences. For example, the commonly used difference scores reflecting the ability to resist interference in the Stroop and flanker tasks, show low reliability (Paap & Sawi, 2016; Paap, Anders-Jefferson, Zimiga, Mason, & Mikulinsky, 2020; Siegrist, 1997; Von Bastian et al., 2016). These measures are also only weakly correlated with each other (Prior et al., 2017; Rey-Mermet, Gade, & Oberauer, 2018; Rouder & Haaf, 2019) even though they are thought to rely on similar processes (Draheim et al., 2020).

The higher variability of mixing costs is not the only possible explanation for why they are more consistent than switching costs. The components of switching costs (stay and switch trials) are more strongly correlated than the components of mixing costs (single and stay trials) and as Draheim, Hicks, and Engle (2016) pointed out, as the correlation between two components increases, the reliability of their difference score decreases.^3^ In Prior and Gollan (2011) for example, the correlation between switch and stay trials in the first administration was 0.95 for the language task and 0.94 for the color-shape task, whereas the correlation between single and stay trials was 0.82 for language and 0.88 for the color-shape task.

### COMPARING RELIABILITIES AND CONSISTENCIES OF THE TASKS TO PREVIOUS STUDIES

The test-retest reliability of the language switching cost in the current study (0.53 when tested twice on the same day and 0.52 when tested a week apart) was lower than that reported in Timmer et al. (2018; 0.74). This difference could be related to differences in the tasks used across studies. Timmer et al. used a trilingual switching task and the linguistic and non-linguistic tasks were administered in the same order over sessions for each participant. In contrast, in our study, two languages were used and in the second session, the language task was administered after the color-shape task, making it susceptible to transfer effects, possibly reducing reliability. Therefore, the interpretation of our findings requires caution. The internal consistency of the language switching costs was also lower than the one reported by Contreras Saavedra et al. (2020; 0.707), who examined the internal consistency across three different conditions (naming single digit numbers, and double-digit numbers).

In contrary, the reliability of color-shape switching costs when tested twice on the same day (0.56) was similar to that of Timmer et al. (0.57) and of Paap and Sawi (0.62), but it was lower (0.40) when tested a week apart. Mixing costs were also less reliable than in Paap and Sawi (0.51 compared to 0.75, respectively). This might also be related to order effects (the color-shape task in our study was administered after the language-switching task in the second session, whereas in Paap and Sawi, the order of tasks was similar in both sessions). Note however that the internal consistency of the color-shape task in the current study was lower than in previous reports by von Bastian and Druey (2017) and von Bastian et al. (2016). While these studies analyzed log transformed and z transformed RT proportional costs, respectively, and used Spearman-Brown coefficient, which provide an estimate of reliability of the test as a whole, we analyzed untransformed RTs. However, even when we used the same procedures as von Bastian, namely proportional costs and Spearman-Brown correlations instead of traditional RTs and Pearson correlations, the same patterns of results emerged – namely lower consistency in the present study (See Tables 2A and 3A in the Appendix).

These cross-study differences might be related to variability. The participants in the current study were more homogeneous (Spanish-English bilinguals in most studies except Prior and Gollan’s study, that also included Chinese-English and Hebrew-English bilinguals), whereas other studies had more variant samples (See Table 1A in the appendix). As we mentioned before, larger variability in true scores is associated with higher reliability (Hedge et al., 2018; Paap and Sawi, 2016). Another possible reason for the difference between the (lower) consistencies of switching costs in the current study compared to the consistencies reported in the past can be related to the relatively small switching costs in our study. For example, the average switching cost in Paap and Sawi (2016) was 201 ms in the first session and 154 ms in the second session, whereas our average switching cost in the first session was 21ms for language switching costs and 28 ms for color-shape switching costs. Larger switching costs make room for more variability, which can increase reliability.

A final possible explanation for cross-study difference can be related to response set. Whereas most studies used manual responses, in the current study all three studies used spoken responses (except for the color-shape task in Stasenko et al.). Spoken responses are more variant than manual responses, but not because of variance in “true score”, but rather because of more error variance (measuring spoken responses is more susceptible to technical errors, such as measuring hesitation at the beginning of a true response). More error variance, as opposed to more variance in true score, can reduce reliability.

### IS THERE AN ALTERNATIVE?

One potential solution for increasing the reliability of switching cost to make them better suited for measuring individual differences is to increase their variability. Variability can be increased by making switching costs larger, either by making the task more difficult (e.g., by switching between 3 languages, and 3 dimensions in the color-shape task, as Timmer et al. did, but without increasing error variability) or by reducing the switching rate, making switching less predictable (Schneider & Logan, 2006). Increasing the number of trials, or imposing response deadlines can also make switching costs more variable or decrease accuracy rates to allow their inclusion in statistical analyses (e.g. Rey-Mermet et al., 2018). Future studies should examine the effect of these manipulations on reliability of switching and mixing costs.

A number of other methodological and statistical alternatives to RTs and RT difference scores have been suggested, assuming that a domain-general switching mechanism does exist and is not manifested in RTs due to methodological shortcomings. By and large, however, these approaches have not successfully increased the reliability of such costs as measures of individual differences. For example, Hughes, Linck, Bowles, Koeth and Bunting (2014) compared the reliability of traditional RT versus accuracy-base switching cost scoring, to other alternatives, which combine RTs and accuracy in a single score in a switching task. In their task, participants judged whether numbers were odd or even versus higher, or lower than 5. Alternative scoring methods examined included rate residual score (the difference between the rate of correct responses per second on switch and stay trials), an inverse efficiency score (dividing RTs by 1minus the percentage of errors) and a bin score (each switch trial RT is subtracted from the participant’s average RT for all stay trials). These residual RTs are then ranked and placed in 10 bins and inaccurate responses are penalized by automatically placing those trials in bad bins, adding additional cost for errors). Accuracy based switching cost (subtracting accuracy rates on stay trials from accuracy rates on switch trials) had the poorest internal consistency and the other measures exhibited levels of internal consistency that were comparable to the latency switching cost. In contrast, Draheim et al. (2016) used the binning procedure in a reanalysis of both their own study (Shipstead et al., 2015) and a different study (Oberauer, Süß, Wilhelm, & Wittman, 2003) and found greater consistency of switching costs than originally reported and more cross-task (working memory and switching costs) correlations. Similarly, Prior, Degani, Awawdy, Yassin, and Korem (2017) also found the binning procedure to increase consistency of switching and mixing costs in bilingual young adults, though in this case the improved consistency did not lead to higher cross-task correlations. The use of the binning procedure may be best suited for large and diverse samples, because it is based on rank-ordered trials. Moreover, individual differences in accuracy are also necessary for the binning procedure to differentiate subjects better than traditional analysis of switching costs (Draheim et al., 2016). In our data sets, accuracy rates were extremely high (above 96%). Therefore, this procedure may not fit language and color-shape switching costs analysis. Lastly, we acknowledge the possible effect of the relatively small sample size on the preciseness and stability of the reliability correlations (Schönbrodt & Perugini, 2013). However, the datasets analyzed in the current study were comparable or even bigger than in previous studies in the field. Therefore, it is representative of what we can expect for previous studies on switching cost correlations.

To sum, language and color-shape task switching and mixing costs are measures commonly used in research on bilingualism, both for group comparisons (e.g., to examine differences between bilinguals and monolinguals in switching and mixing abilities) and for studies of individual differences (e.g., comparing bilinguals to monolinguals, or switching and mixing costs across domains). The present study demonstrated that the language-switching task is as reliable and as consistent as the color-shape task in measuring switching costs, and the language task was more consistent than the color-shape task in measuring mixing costs. We also suggest that variability in true score affects the reliability and consistency of switching and mixing costs. Critical differences between tasks might reflect the use of voice responses in the language task, or the inherently sequential nature of bilingual language use as an instance of multi-tasking, versus simultaneous processing of color and shape dimensions. These factors require further investigation and should be considered when using switching and mixing costs to address questions regarding the specificity or domain-generality of cognitive flexibility in multi-tasking and of switching between sequential tasks.

## DATA ACCESSIBILITY STATEMENT

The data analyzed in this study is available online. See osf.io/4A895 for Prior and Gollan (2013) and for Stasenko et al. (2017) and see osf.io/zcb52 for Kleinman and Gollan (2018).

## ADDITIONAL FILE

The additional file for this article can be found as follows:

10.5334/joc.140.s1 Appendix. Tables 1A, 2A and 3A.

## Footnotes

In our reanalysis of RT data from Prior & Gollan (2013), we removed errors (2% from each task), trials after errors (2% from each task), RTs < 250 (less than 1% from each task), and RTs > 3000 (less than 1% from each task). After removing these trials, following Paap and Sawi (2016), RTs that were more than 2.5 SDs from each participant’s mean were also trimmed for each task separately, across sessions and conditions; (2% of trials in language switching task and 5% of trials in color-shape task). Two participants in the language and two in the color-shape group had less than 60% of trials in at least one of the conditions in one of the sessions and were removed from the analysis. Similarly, in our reanalysis of Stasenko, Matt, & Gollan (2017) data, we removed errors (2% in each task), trials after errors (2% in each task), RTs < 250 ms (less than 0.5% in each task) and trials > 3000 (2% in each task). We also removed RTs above 2.5 SDs (6% of short and 7% of long CTI in the language task and 7% from short and long CTIs in color-shape task) and below 2.5 SDs (less than 0.5% short and long CTI in the language task and no trials in short and long CTI in color-shape task) from each subjects’ mean. Two participants did not complete the mixed blocks of the language task and were removed from the analyses. In our reanalysis of data from Kleinman and Gollan (2018), we removed errors (4% of trials), trials following errors (4%), RTs < 250 ms (less than 0.5%) and RTs > 3000 ms which were coded as “no response” (4%). We also removed RTs above 2.5 SDs (3%) or below 2.5 SDs (less than 0.1%) of each subject’s mean. Note that the trimming procedures in this reanalysis, were somewhat different from the original trimming procedures to allow better comparison across the 3 data sets and comparison to other studies in the field (e.g., Paap and Sawi, 2016). This resulted in different number of participants in Prior and Gollan and in slightly different number of trials in all 3 data sets.

To allow better comparison across studies, switching and mixing costs were also calculated as proportional costs (switching and mixing costs were divided by the average RT of the trials involved; Timmer et al., 2018). Importantly, this did not change the pattern of results (See Tables 2A and 3A in the Appendix).

The formula estimating reliability of difference score, given by Guilford (1954) and Lord (1963) and cited in Draheim et al. (2016) is:

Pdd^\prime\ = \frac{{Pxx^\prime\ - Pxy}}{{1 - Pxy}}

*Pdd*′ is estimated reliability of the difference score, *Pxx*′ is estimated reliability of the two component scores, and *Pxy* is correlation between the two component scores.
