# Supplementary material for: Do All Switches Cost the Same? Reliability of Language Switching and Mixing Costs
Source: J Cogn. 2021 Jan 7;4(1):3. doi: 10.5334/joc.140 (PMC7792451; doi:10.5334/joc.140)
Supplement: Appendix. — Tables 1A, 2A and 3A. [file joc-4-1-140-s1.pdf]

Appendix

Table 1A

Review of previous reports of reliability and consistency of non-linguistic switching costs

	**Timmer at al. (2018)**	**Paap and Sawi (2016)**	**Von Bastian et al. (2016)**	**von Bastian and Druey (2017)**
Task	Perceptual classifications: color, size and type (letter vs. number)	Color-shape	Color-shape<br>Animacy-size<br>Parity-magnitude	Color-shape
Reliability type	Test-retest (5–9 days)	Test-retest (week)	Split half	Split half
Reliability measure	Cronbach's alpha (ICC)<br>*Switching r = 0.57*	Probably Pearson<br>*Switching r = 0.62*<br>*Mixing r = 0.74*	Spearman Brown<br>*Switching r = .91*<br>*Mixing r = 0.96*<br>Averaged across tasks	Spearman Brown<br>*Switching r = 0.79*
Switching and mixing cost calculation	proportional cost:<br>Switch cost x 100<br>Average- stay and switch	Probably standard	Proportional cost<br>Dependent variables were z-transformed"	Log transformed switch costs
Participants	53 Trilinguals	75 Bilinguals and monolinguals	118 bilinguals (various language combinations)	120 (do not mention if bilinguals or not)
Tasks order	Same order in both sessions	Same order in both sessions	One session	One session
Number of trials	~ 70 stay and 250 switch trials	72 stay and 72 switch trials	64 stay and 64 switch	72 stay and 72 switch
Response type	Manual	Manual	Manual	Manual

Table 2A
Test-retest reliability of single, stay and switch trials and of switching and mixing costs in the language switching task in Prior and Gollan (2013) and internal consistency (correlations between even and odd trials) of the language switching task in Prior and Gollan and Stasenko et al. (proportional switching and mixing scores calculated for Pearson and Spearman Brown correlations in grey).

	Test-retest		Internal consistency			
	Prior and Gollan (2013)		*Prior and Gollan (2013)*		*Stasenko et al. (2017)*	
	same day	over a week	1st session [a]	transfer task [b]	CTI Long	CTI Short
Single	0.92	0.87	0.98	0.97	0.96	0.97
Stay	0.93	0.88	0.93	0.96	0.97	0.97
Switch	0.92	0.82	0.95	0.96	0.96	0.97
switching cost	0.53	0.52	0.32	0.45	0.37	0.41
SC_Proportional (P)	0.52	0.49	0.26	0.39	0.37	0.33
SC_Proportional (S)	0.46	0.43	0.25#	0.31	0.45	0.41
mixing cost	0.79*	0.67	0.77*	0.79*	0.89*	0.87*
MC_Proportional (P)	0.74	0.62	0.75	0.75	0.85	0.83
MC_Proportional (S)	0.60	0.53	0.64	0.82	0.85	0.82

SC = Switching costs, MC = mixing costs, (P) = Pearson correlation, (S) = Spearman-Brown correlation. [a] First administration of the task on the first day. [b] Administration of the task after training on the other task # n.s

Table 3A
Test-retest reliability of single, stay and switch trials and of switching and mixing costs in the color-shape switching task in Prior and Gollan (2013) and internal consistency (correlations between even and odd trials) of the color-shape switching task in Prior and Gollan and Stasenko et al. (proportional switching and mixing scores calculated for Pearson and Spearman Brown correlations in grey)

	Test-retest		Internal consistency			
	Prior and Gollan (2013)		Prior and Gollan (2013)		Stasenko et al. (2017)	
	same day	over a week	1st session [a]	transfer task [b]	CTI Long	CTI Short
Single	0.92	0.87	0.97	0.97	0.96	0.95
Stay	0.88	0.82	0.92	0.94	0.96	0.97
Switch	0.90	0.76	0.91	0.95	0.96	0.97
switching cost	0.56	0.40	0.14#	0.29	0.17#	0.43
SC_Proportional (P)	0.54	0.45	0.18#	0.30	0.19#	0.42
SC_Proportional (S)	0.48	0.36	0.17#	0.37	0.22#	0.41
mixing cost	0.53	0.51	0.70*	0.82*	0.91*	0.91*
MC_Proportional (P)	0.54	0.44	0.66	0.81	0.89	0.86
MC_Proportional (S)	0.50	0.51	0.62	0.78	0.88	0.85

SC = Switching costs, MC = mixing costs, (P) = Pearson correlation, (S) = Spearman-Brown correlation. [a] First administration of the task on the first day. [b] Administration of the task after training on the other task # n.s
